# Supplementary material for: Phylogeny and divergence times of suckers (Cypriniformes: Catostomidae) inferred from Bayesian total-evidence analyses of molecules, morphology, and fossils
Source: PeerJ. 2018 Jul 4;6:e5168. doi: 10.7717/peerj.5168 (PMC6035723; doi:10.7717/peerj.5168)
Supplement: Supplemental Information 1 [file peerj-06-5168-s001.docx]

**Appendix S1.** Catostomidae fossils, fossil and external calibration age priors, and additional discussion of phylogenetic relationships within the Catostominae.

**Table of Contents**

**1. Introduction 1**

**2. Review: Sucker Fossils 2**

**3. Calibrations Used for Sucker Divergence Dating 16**

**4. Unsuitable Minimum Age Calibrations for Suckers 16**

**5. Additional Phylogenetic Relationships Within the Catostominae 17**

**6. References 19**

**1. Introduction**

It is important to use objective and repeatable methods for identifying minimum age justifications for fossil sampling dates or calibrations used in phylogenetic studies. By following published recommendations in Parham et al. (2012) and precedents set by previous empirical studies (e.g. Pyron, 2011; Near et al., 2011, 2012; Unmack et al., 2014), we were able to identify a suitable external calibration for our sucker tree based on a phylogenetic study. Subsequently, we used this external calibration to set the time of origin prior (*t*_or_) for the fossilized birth-death (FBD) process tree prior (Gavryushkina et al., 2013; Heath et al., 2014) that we used in all of our BEAST relaxed-clock divergence time analyses of sucker diversification in this study. Additionally, we were able to include dates for 19 extinct sucker taxa in our FBD analyses, most of which were derived from fossil information, with the only exception being the age assigned to the historically extinct species †*Moxostoma lacerum* (see discussion in main text; this species is omitted here because there are no fossil records of it).

In this Appendix, our objectives are four-fold. First, we present the results of a literature review of the sucker fossil record that we conducted considering 33 sucker fossils from Asia and North America. We present a table summarizing the taxonomy of extinct fossil sucker species that we are aware have been described to date. Also in table format, we review the spatial and temporal distributions of sucker fossils, while highlighting minimum age calibrations that we estimate can be obtained from each fossil, and attending geological and phylogenetic metadata. Our fossil review is not necessarily exhaustive, but it covers the most well-known and best-justified fossil specimens for the family, while excluding questionable fossils. It is worth reiterating that this review provided the raw data for determining suitable fossil ages and prior settings used in our BEAST analyses.

Second, we provide background information and justifications for the external root-age calibration point we used in our FBD BEAST runs mentioned above. We list details on this calibration, including characters and phylogenetic results on which the calibration is based, the minimum age estimate, and prior settings supplied to BEAST. Then, we list (1) the calibrated node, (2) the taxa that represent the first known occurrence of the lineage in the fossil record, and (3) available phylogenetic results supporting the clade in question, or linking the fossil to the group and aiding its placement in the sucker phylogeny. We also list (4) the location and age of the geological formation(s) for the fossil assigned to the node, as well as (5) our minimum age assessment based on the available phylogenetic and geological data. Calibration ID letters and numbers referred to here are the same as those given in the text.

Third, we briefly review why some of the fossils for which we were able to determine minimum age justifications were not used in our BEAST analyses.

Fourth, we here provide a supplemental presentation and discussion of the finer-scale patterns of relationships within and among clades 1–9 that we resolved for the subfamily Catostominae (e.g. Figs. 3 and S4). We present this material here due to space constraints: including this material in the main text would have rendered the Results and Discussion section too long, making it burdensome for the reader. In doing so, we avoid this issue while keeping the material peer-reviewed to ensure objectivity of presentation. However, in order to avoid duplicating content, we mainly present information here on clades that were not emphasized in Section 3.4 of the main text.

**2. Review: Sucker Fossils**

The first fossil sucker genus and species, †*Amyzon mentale* Cope 1872, was described by Cope (1872) from North American fossil beds at the Osino Oil Shales of Nevada, which date to ~37–34 million years ago (Ma) in the late Eocene–early Oligocene (Bruner, 1991; Swisher & Prothero, 1990). Since Cope’s groundbreaking systematic work on fossil suckers nearly 150 years ago, contributions from many workers clearly demonstrate that suckers possess a rich and relatively complete fossil record, especially when compared to other speciose North American freshwater fish clades for which little or no fossil information is available (e.g. darters within family Percidae; reviewed by Smith, 1981; Cavender, 1986, 1998; Smith et al., 2002). Presently, sucker fossils span a wide geographical area, ranging in Asia from Mongolia to central–eastern China and eastern Siberia, and dotting the western North American continent from British Columbia to Mexico, and from inland California to eastern Texas (e.g. Cavender, 1998: Fig. 3; Chang et al., 2001: Fig. 1). The sucker fossil record also covers a wide timespan, with fossilized specimens ranging in age from the Eocene up until the end-Pleistocene (~56–0.01 Ma). Thus, the available fossils demonstrate that suckers have been distributed over large areas in multiple Holarctic infraregions in at least western North America and eastern Asia throughout the Cenozoic, and that major ‘derived’ lineages of suckers (e.g. genera within subfamily Catostominae) having been undergoing speciation since at least the Miocene (Smith, 1975, 1981, 1992; Cavender, 1986, 1998).

For additional information on North American sucker fossils, we refer the reader to broad summaries comparing Catostomidae against other North American or European freshwater fish families (Smith, 1981; Cavender, 1986, 1998; Smith et al., 2002, 2013), as well as the recent, comprehensive review of the biogeography, systematics, ecology, and natural history of extant and fossil suckers by Harris et al. (2014). The taxonomy of North American fossil suckers has been relatively stable for decades, with the principal disagreements being the fluctuating number of species within †*Amyzon* (Grande et al., 1982; Bruner, 1991), and that some authors have recognized the elevation of subgenus *Pantosteus* to genus status (Minckley, 1973; Unmack et al., 2014), while others have not (Smith, 1966, 1992; Smith & Koehn, 1971; Harris & Mayden, 2001; Harris et al., 2002; Smith et al., 2013). Compared with North American specimens, the Asian fossil sucker assemblage is lesser known and represented by fewer collections, and it has traditionally exhibited more taxonomic confusion and instability, for example due to the use of some fragmentary bone material and inadequate comparisons with North American material (Chang & Chow, 1986; Chang et al., 2001). However, several reviews as well as empirical studies of fossil sucker systematics and biogeography provide excellent sources of information on the Asian fossil specimens, which include the most recent fossil sucker species to be described or redescribed (Chang & Chow, 1986; Sytchevskaya, 1986; Smith, 1992; Chang et al., 2001; Chang & Chen, 2008; Liu & Chang, 2009; Harris et al., 2014).

Fossil suckers have been noted, or formally taxonomically described, for both extant and extinct lineages of suckers in North America and Asia (Tables A1 and A2). Among the 15 species of extinct fossil suckers described from North American sites that we include here, there are 12 currently valid species, and these are assignable to the following five genera (subfamilies in parentheses): †*Amyzon* Cope 1872 (Ictiobinae), *Catostomus* Lesueur 1817 (Catostominae), *Chasmistes* Jordan 1878 (Catostominae), *Ictiobus* Rafinesque 1820 (Ictiobinae), and *Moxostoma* Rafinesque 1820 (Catostominae) (Table A1). While all 12 of these species are endemic to North America, no extinct fossil genera are endemic to the continent. The only extinct genus of suckers found in North America is †*Amyzon*, which is known from productive fossil beds of British Columbia, Washington, Nevada, and Wyoming (Table A2). Aside from †*Amyzon*, all North American sucker fossils are believed to correspond to extant lineages; indeed, fossils are available for seven extant sucker species, including *Ictiobus cyprinellus*, *I. niger*, *Catostomus ardens*, *C. commersoni*, *C. discobolus*, *Moxostoma duquesnei*, and *Xyrauchen texanus* (Table A2). We note that several fossil sucker collections from North America whose bones could possibly be placed with other species are not reviewed here. For example, we exclude “†*Deltistes ellipticus*” maxilla bones of Miller & Smith (1967) that were placed with †*Catostomus shoshonensis* material by Smith et al. (1982), and dentaries attributed to “†*Catostomus cristatus*” by Smith (1975) that were possibly identifiable as †*C. shoshonensis* (Smith et al., 1982).

Three genera and species of fossil suckers that have been described from Asia are discussed here, all of which are extinct (Table A1). Two of these taxa are widely agreed upon as valid and having clear morphological affinities or differences with other suckers; these are †*A. huanensis* (Cheng 1962) and †*Plesiomyxocyprinus arratiae* Liu & Chang 2009 (Table A1). A third taxon, the Eocene–Oligocene genus †*Vasnetzovia*, was described by Sytchevskaya (1986) from complete specimens collected north of Vladivostok, Primorsky Krai in eastern Siberia, Russia. †*Vasnetzovia* is also considered validly described, and is monotypic with one species, †*V. artemica* Sytchevskaya 1986 (e.g. Chang & Chen, 2008; Harris et al., 2014). However, we exclude from our analysis the first fossil sucker known from Asia, which was described from disarticulated opercles and vertebrae sampled from middle Eocene rocks of the Gobi Desert, Inner Mongolia, and identified as “*Catostomus*” (Hussakof, 1932). We exclude this record because Nelson (1949) showed that this specimen was misidentified as belonging to genus *Catostomus* and instead may be referable to another sucker genus, either *Carpiodes* or *Myxocyprinus*, or might not even be a catostomid. We also exclude records of 10 Asian fossil sucker species from Eocene–Oligocene deposits of Kazakhstan, Mongolia, and Siberia that Sytchevskaya (1986) described as belonging to †*A. gosiutensis* (a species only known from North America), two new species of †*Amyzon* (†*A. interruptus*, and †*A. zaissanicus* from the Zaissan Basin of eastern Kazakhstan), and seven new species allocated to extant sucker genera (*Carpiodes brevidens*, *Cycleptus robustus*, *Catostomus columnaris*, *Erimyzon luxus*, *Minytrema shevyrevi*, *Moxostoma fungidens*, and *Xyrauchen rotundus*). We exclude these taxa because they were described based on isolated specimens of pharyngeal teeth samples, without the context of complete specimens or adequate comparisons to North American material (Sytchevskaya, 1986). Smith (1992) dismissed all of Sytchevskaya’s species above based on pharyngeal teeth because he deemed the original materials as lacking sufficiently detailed information to warrant the descriptions she authored. Moreover, Smith (1992) argued that the Asian *Cycleptus* species Sytchevskaya proposed was based on misidentified materials when compared with Eastman (1977), and that her Asian *Carpiodes* specimen from Kazakhstan was insufficient to diagnose the form as being distinct from †*Amyzon*.

**Table A1.** Summary of the taxonomy and distributions of extinct sucker (Catostomidae) species known from the fossil record.

| No. | Described fossil species | Subfamily (Tribe) | Status | Geological epoch | Geographical distribution | Fossil references |
| --- | --- | --- | --- | --- | --- | --- |
| 1 | †*Amyzon aggregatum* (Wilson 1977) | Ictiobinae | Valid (Bruner, 1991) | Early Eocene | British Columbia, Pacific northwestern North America | Wilson (1977); Grande et al. (1982); Bruner (1991) |
| 2 | †*A. brevipinne* (Cope 1893) | Ictiobinae | Valid (Bruner, 1991) | Early Eocene | British Columbia, Pacific northwestern North America | Cope (1893); Grande et al. (1982); Bruner (1991) |
| 3 | †*A. commune* (Cope 1874) | Ictiobinae | Valid (Bruner, 1991) | Late Eocene | Colorado, western North America | Cope (1874); Bruner (1991) |
| 4 | †*A. fusiforme* Cope 1875 | Ictiobinae | Junior synonym of *A. commune* (Bruner, 1991) | Eocene | Colorado, western North America | Cope (1875); Bruner (1991) |
| 5 | †*A. gosiutensis* Grande, Eastman, & Cavender 1982 | Ictiobinae | Junior synonym of *A. aggregatum* (Bruner, 1991) | Early middle Eocene | Wyoming, western North America | Grande et al. (1982); Bruner (1991) |
| 6 | †*A. huanensis* (Cheng 1962) | Ictiobinae | Valid, described by Chang et al. (2001) based on older materials that were incorrectly referred to the cyprinid genus *Osteochilus* by Cheng (1962) | Eocene | Hunan Province, southern China | Cheng (1962); Chang et al. (2001) |
| 7 | †*A. mentale* (Cope 1872) | Ictiobinae | Valid (Bruner, 1991) | Eocene–Oligocene | Nevada, western North America | Cope (1872); Bruner (1991) |
| 8 | †*A. pandatum* Cope 1874 | Ictiobinae | Junior synonym of *A. commune* (Bruner, 1991) | Eocene | Colorado, western North America | Cope (1874); Bruner (1991) |
| 9 | †*Ictiobus aguilerai* Alvarado-Ortega et al. 2006 | Ictiobinae | Valid (Alvarado-Ortega et al., 2006) | Pliocene | Hidalgo, central Mexico | Alvarado-Ortega et al. (2006) |
| 10 | †*Vasnetzovia artemica* Sytchevskaya 1986 | Ictiobinae | Valid (Chang & Chen, 2008; Harris et al., 2014) | Eocene–early Oligocene | eastern Siberia, northeastern Russia | Sytchevskaya (1986) |
| 11 | †*Plesiomyxocyprinus arratiae* Liu & Chang 2009 | Myxocyprininae | Valid (Liu & Chang, 2009) | Early middle Eocene | various Huadian Formation coal mines, Jilin Province, northeastern China | Liu & Chang (2009) |
| 12 | †*Catostomus arenatus* Miller & Smith 1967 | Catostominae (Catostomini) | Valid (Smith et al., 1982) | Late Miocene–Pliocene | Idaho, western North America | Miller & Smith (1967); Smith (1975); Taylor & Smith (1981); Smith et al. (1982, 2013) |
| 13 | †*Catostomus asitus* Smith, Steward, & Carpenter 2013 | Catostominae (Catostomini) | Valid (Smith et al., 2013) | Early Pliocene | Nevada, western North America | Smith et al. (2013) |
| 14 | †*Catostomus hyomyzon* Smith, Steward, & Carpenter 2013 | Catostominae (Catostomini) | Valid (Smith et al., 2013) | Miocene | Oregon, western North America | Smith et al. (2013) |
| 15 | †*Catostomus oromyzon* Smith, Steward, & Carpenter 2013 | Catostominae (Catostomini) | Valid (Smith et al., 2013) | Pliocene | Idaho, western North America | Smith et al. (2013) |
| 16 | †*Catostomus owyhee* (Miller & Smith 1967) | Catostominae (Catostomini) | Valid (Smith, 1975; Smith et al., 2013); originally described as “*Deltistes owyhee*” by Miller & Smith (1967) | Late Miocene–Pliocene | Idaho, western North America | Miller & Smith (1967); Smith (1975) |
| 17 | †*Catostomus shoshonensis* Cope 1883 | Catostominae (Catostomini) | Valid (Smith et al., 1982, 2002) | Late Miocene–Pliocene | Idaho, western North America | Cope (1883); Armstrong et al. (1975); Smith (1975); Taylor & Smith (1981); Smith et al. (1982) |
| 18 | †*Chasmistes spatulifer* (Miller & Smith 1967) | Catostominae (Catostomini) | Valid (Smith, 1975; Smith et al., 2002) | Late Miocene–Pliocene | Idaho, western North America | Miller & Smith (1967); Smith (1975) |

Abbreviations: Co., county; No., number.

**Table A2.** Summary of Catostomidae fossils and potential derived minimum age justifications for phylogenetic fossil calibrations.

| No. | Fossil taxon | Age (Ma) | Gap analysis age at node (Ma) | Minimum age justification (Ma) | Location | Fossil references | Age references | Minimum age references |
| --- | --- | --- | --- | --- | --- | --- | --- | --- |
| 1 | †*Amyzon aggregatum* | 49.42 ± 0.54, Ypresian-Lutetian or Bridgerian NALMA | – | 48.88 | Klondike Mountain Formation, British Columbia | Wilson (1977); Grande et al. (1982); Greenwood et al. (2005) | Wolfe et al. (2003) | Wolfe et al. (2003) |
| 2 | †*A. aggregatum* (*Amyzon gosiutensis*) | 48–45, early Middle Eocene (Bridgerian) | – | 47.09 | Laney Shale Member of the Green River formation, Wyoming | Grande et al. (1982); Bruner (1991) | Mauger (1977); Smith et al. (2010) | Smith et al. (2010) |
| 3 | †*A. brevipinne* | 49.42 ± 0.54, Ypresian-Lutetian or Bridgerian NALMA | – | 48.88 | Klondike Mountain Formation, British Columbia | Wilson (1977); Grande et al. (1982); Greenwood et al. (2005) | Wolfe et al. (2003) | Wolfe et al. (2003) |
| 4 | †*A. commune* | 34.7–33.7 Ma, Late Eocene–Early Oligocene | – | 33.7 | Florissant Formation, South Park, Colorado | Wilson (1977); Cope (1874); Bruner (1991) | Bruner (1991) | Prothero & Swisher (1992); Prothero & Sanchez (2004) |
| 5 | †*A. huanensis* | 47.8–37.8, Middle Eocene (taken as Lutetian to Bartonian) | – | 37.8 | Xiawanpu, Xiangxiang, Hunan Province, southern China | Cheng (1962); Chang et al. (2001) | Chang et al. (2001) | Walker et al. (2012) |
| 6 | †*A. mentale* | 37–34, Late Eocene–early Oligocene, Chadronian-Orellan NALMA | – | 34.0 | Osino Oil Shales, near Elko, Nevada | Cope (1872); Bruner (1991) | Bruner (1991); Cavender (1986, 1998); Harris et al. (2014) | Swisher & Prothero (1990) |
| 7 | †*Ictiobus aguilerai* | Pliocene  Blancan | – | 1.72 | Tula de Allende, Hidalgo, Mexico | Alvarado-Ortega et al. (2006) | Alvarado-Ortega et al. (2006) | Bell et al. (2004) |
| 8 | *Ictiobus cyprinellus* | 4.1–3.4^a^, Pliocene | 7.2 | 3.4 | Sand Draw local fauna, Brown Co., Nebraska | Smith & Lundberg (1972) | Bell et al. (2004); Near et al. (2005) | Bell et al. (2004); Near et al. (2005) |
| 9 | *Ictiobus niger* | Pleistocene Sheridanian | – | 0.3 | Kanopolis local fauna, Ellsworth Co., Kansas | Neff (1975) | Neff (1975); Repenning (1987) | Neff (1975); Repenning (1987) |
| 10 | *Ictiobus* sp. | Pleistocene Sangamonian interglacial | – | 0.055 | Lower Shuler member of the Pemberton Hill-Lewisville (T2 terrace) of the Trinity River, Dallas Co., Texas | Uyeno & Miller (1962) | Uyeno & Miller (1962); Markewich et al. (2011) | Uyeno & Miller (1962); Markewich et al. (2011) |
| 11 | †*Ictiobus* sp. cf. *bubalus* | 10–9.5 Ma, Tortonian (Miocene; Walker et al., 2012) | – | 9.5 | Laverne local fauna, Beaver Co., Oklahoma | Smith (1962) | Smith (1962); Tedford et al. (2004) | Tedford et al. (2004) |
| 12 | *Ictiobus* sp. | Pleistocene Illinoian glaciation | – | 0.135 | Mt. Scott fauna, Meade Co., Kansas | Smith (1963) | Hibbard (1963); Wood et al. (2010) | Wood et al. (2010) |
| 13 | *Ictiobus* sp. | 12.0^a^; medial Clarendonian | – | 10.0 | Wakeeney local fauna, Trego Co., Kansas | Wilson (1968) | Wilson (1968); Tedford et al. (1987, 2004) | Woodburne (2007) |
| 14 | †*Vasnetzovia artemica* | 37.8–28.1, Late Eocene–early Oligocene (taken as Priabonian to Rupelian) | – | 28.1 | Wuglovaya Formation, Artem lignite mine, Primorsky Krai, Russia | Sytchevskaya (1986) | Sytchevskaya (1986); Liu & Chang (2009) | Walker et al. (2012) |
| 15 | †*Plesiomyxocyprinus arratiae* | 39.74 ± 0.07, Early middle Eocene; correlated to the late Uintan NALMA | – | 39.67 | Huadian Formation, Jilin Province, China | Liu & Chang (2009) | Wang & Li (1990) | Prothero & Swisher (1992) |
| 16 | *Catostomus ardens* | 0.0112–0.01 Ma, end-Pleistocene–Holocene | – | 0.01 | Homestead Cave, Box Elder Co., Utah | Broughton (2000) | Broughton (2000) | Broughton (2000) |
| 17 | †*Catostomus arenatus* | 2.4–1.9 | – | 1.9 | Pliocene Sand Point local fauna, Glenns Ferry Formation, Idaho | Miller & Smith (1967); Smith (1975); Smith et al. (1982, 2013) | Miller & Smith (1967); Smith et al. (2013) | Miller & Smith (1967); Smith et al. (2013) |
| 18 | †*Catostomus asitus* | 4.7–4.5 | – | 4.5 | Starvation Flat area, Pliocene White Narrows formation, southern Nevada | Smith et al. (2013) | Schmidt et al. (1996); Reynolds & Lindsay (1999) | Schmidt et al. (1996); Reynolds & Lindsay (1999) |
| 19 | *Catostomus columbianus* | 2.4–1.9 | – | 1.9 | Pliocene Sand Point local fauna, Glenns Ferry Formation, Idaho | Miller & Smith (1967); Smith (1975); Smith et al. (1982, 2013) | Miller & Smith (1967); Smith et al. (2013) | Miller & Smith (1967); Smith et al. (2013) |
| 20 | *Catostomus commersoni* | Irvingtonian Land Mammal Age | – | 0.160 | Berends Fauna, Beaver Co., Oklahoma | Smith (1954) | Smith & Cifelli (2000) | Bell et al. (2004) |
| 21 | *Catostomus commersoni* | Pleistocene Illinoian glaciation | – | 0.135 | Mt. Scott fauna, Meade Co., Kansas | Smith (1963) | Hibbard (1963); Wood et al. (2010) | Wood et al. (2010) |
| 22 | *Catostomus discobolus* | 0.0112–0.01 Ma, end-Calabrian Pleistocene | – | 0.01 | Homestead Cave, Box Elder Co., Utah | Broughton (2000) | Broughton (2000) | Broughton (2000) |
| 23 | †*Catostomus hyomyzon* | 11.6 | – | 12.7^b^ | Juntura Formation, Malheur Co., Oregon | Smith et al. (2013) | Smith et al. (2013) | Unmack et al. (2014) |
| 24 | †*Catostomus oromyzon* | 4.5, late Miocene | – | 5.5^b^ | Glenns Ferry Formation, Owyhee Co., Idaho | Smith et al. (2013) | Smith et al. (2013) | Unmack et al. (2014) |
| 25 | †*Catostomus owyhee*^c^ | 4.4–6.2, late Miocene–Pliocene | – | 3.0 | Glenns Ferry fauna, multiple counties, Idaho | Miller & Smith (1967); Smith (1975) | Armstrong et al. (1975); Ruez & Ginsler (2008) | Ruez & Ginsler (2008) |
| 26 | †*Catostomus shoshonensis* | 4.4–6.2, late Miocene–Pliocene | – | 3.0 | Glenns Ferry fauna, multiple counties, Idaho | Cope (1883); Armstrong et al. (1975); Smith (1975); Taylor & Smith (1981); Smith et al. (1982) | Armstrong et al. (1975); Ruez & Ginsler (2008) | Ruez & Ginsler (2008) |
| 27 | †*Catostomus* sp. cf*. tahoensis* | Pliocene | – | 2.588 | Mopung Hills, Churchill Co., Nevada | Taylor & Smith (1981) | Taylor & Smith (1981) | Taylor & Smith (1981); Gibbard et al. (2009); Walker et al. (2012) |
| 28 | *Catostomus* sp. | Pliocene | – | 2.588 Ma | Honey Lake, Lassen Co., California | Taylor & Smith (1981) | Taylor & Smith (1981) | Taylor & Smith (1981); Gibbard et al. (2009); Walker et al. (2012) |
| 29 | †*Chasmistes* sp. cf. *cujus* | Pliocene | – | 2.588 Ma | Honey Lake, Lassen Co., California | Taylor & Smith (1981) | Taylor & Smith (1981) | Taylor & Smith (1981); Gibbard et al. (2009); Walker et al. (2012) |
| 30 | †*Chasmistes spatulifer* | 4.4–6.2, late Miocene–Pliocene | – | 3.0 | Glenns Ferry fauna, multiple counties, Idaho | Miller & Smith (1967); Smith (1975) | Armstrong et al. (1975); Ruez & Ginsler (2008) | Ruez & Ginsler (2008) |
| 31 | *Moxostoma duquesnei* | Pleistocene Illinoian glaciation | – | 0.135 | Mt. Scott fauna, Meade Co., Kansas | Smith (1963) | Hibbard (1963); Wood et al. (2010) | Wood et al. (2010) |
| 32 | *Moxostoma* sp. | Miocene–Pliocene Blancan | – | 1.72 | Lake Chapala local fauna, Jalisco, Mexico | Smith et al. (1975) | Israde-Alcántara et al. (2010) | Bell et al. (2004) |
| 33 | *Xyrauchen texanus* | 5.3–3.6 Ma, Early Pliocene | – | 3.6 | Anza-Borrego Desert, San Felipe Hills, Imperial Co., California | Stewart & Roeder (1993); Hoetker & Gobalet (1999) | Stewart & Roeder (1993) | Stewart & Roeder (1993) |

^a^ From Near et al. (2005).

^b^ Corrected age for stem lineage branch based on method of Marshall (1990).

^c^ Probably the sister lineage to *Deltistes*, a monotypic genus containing the species *D. luxatus*.

All fossil data are from localities within the United States, unless stated otherwise. Abbreviations: Co., county; Ma, millions of years ago; NALMA, North American Land Mammal Age; No., number; sp., species.

**3. Calibrations Used for Sucker Divergence Dating**

**Root (R).** *Node*: root node of tree containing five cypriniform outgroup taxa plus Catostomidae ingroup in our analysis. Catostomidae is placed within Cypriniformes, with parent taxon Cyprininae, by Betancur-R et al. (2013). *Character states*: catostomids share various similarities with other cypriniform groups indicating correct placement together in the order, as well as possible sister group relationships; among these, suckers share joined second and fourth pleural ribs of the Weberian apparatus with *Gyrinocheilus*; a single row of pharyngeal teeth and reduced first basibranchial with Cobitidae fishes; and lobed epibranchials, paired basipterygoid processes articulated with the first branchial arches, and enlarged, triangular supraneurals completely fused into the expanded neural complex of the Weberian apparatus with the cyprinids *Cyprinus* and *Carassius* (reviewed by Smith, 1992). *Resolution in phylogenetic analysis*: Smith’s (1992) analysis of 157 mostly morphological (but also biochemical and transition series) characters resolved Catostomidae as monophyletic relative to two cypriniform outgroup lineages, *Cyprinus* (Cyprinidae) and *Leptobotia* (Cobitidae). More recently, a phylogenetic analysis of a dataset of nine nuclear genes with a total of 7587 DNA nucleotides in the matrix resolved two species of Catostomidae in a Cypriniformes clade also containing two lineages of minnow fishes (Cyprinidae: *Danio*, *Opsariichthys*), with definitive (100%) maximum-likelihood bootstrap support (Near et al., 2012). Catostomidae is also placed in Cypriniformes along with members of the families Botiidae, Cobitidae, Cyprinidae, Gyrinocheilidae, and Nemacheilidae with high (100%) bootstrap support, based on phylogenetic analysis of 21 mitochondrial and nuclear genes for 1410 bony fish taxa by Betancur-R et al. (2013). *Absolute age estimate*: 94.9 Ma with Bayesian credible intervals of 113.3–78.9 Ma (Near et al., 2012). *Prior setting*: a normal distribution with the mean = 94.9, sigma = 9.0, and offset = 0. The mean and bounds are based on the *t*_MRCA_ for Cypriniformes estimated by Near et al. (2012) using fossil-calibrated molecular divergence time analyses.

**4. Unsuitable Minimum Age Calibrations for Suckers**

Fossils should not be used as point estimates, because the oldest fossil available for a lineage will always underestimate the age of the true point of origin of that lineage; instead, fossils should be treated as minimum age estimates (Ho & Phillips, 2009; Parham et al., 2012). As shown above, we arrived at several reasonable minimum age priors, and some soft and hard upper bound priors, for our analyses of sucker diversification based on a synthesis of paleontological, geological, and phylogenetic information. However, given the fossil record is better for some sucker lineages (e.g. subfamily Ictiobinae, tribe Catostomini) than others (e.g. subfamily Cycleptinae or tribe Moxostomatini), and that there is tamphonomic bias in the record favoring the preservation of specimens from lacustrine or palustrine environments (Elder & Smith, 1988), fossils from relatively recent deposits (e.g. dating to <1 Ma or <0.5 Ma) should not be used to place priors on divergence times because they might vastly underestimate the timing of lineage divergence. These kinds of fossils are deceptive, because they are young in age, but they can represent lineages that have existed for several or many millions of years. In the case of the suckers, the fossils that appear most likely to underestimate the timing of lineage divergence include (1) the end-Pleistocene *C. ardens* and *C. discobolus* fossil from Homestead Cave (0.0112–0.01 Ma; Broughton, 2000), (2) late Pleistocene *C. commersoni* fossils dated to the Irvingtonian age and Pleistocene Illinoian glaciation (0.16–0.135 Ma; Smith, 1954, 1963), (3) *Ictiobus* fossils from the mid-late Pleistocene Sheridanian and Sangamonian (Neff, 1975; Uyeno & Miller, 1962), and (4) the Pleistocene Illinoian-aged *Moxostoma duquesnei* fossil from Mt. Scott fauna (Smith, 1963). As a result, we did not include these fossils in our divergence dating analyses. Other fossils reviewed above are not necessarily likely to underestimate the age of particular lineages because they are from very recent strata, but were deemed unsuitable because older age estimates were available from other fossils from the same lineage. For example, phylogenetic hypotheses indicate that the *Catostomus* subgenus *Pantosteus* forms a monophyletic lineage except for *C.* (*P.*) *columbianus* (Smith, 1992; Unmack et al., 2014), and the oldest fossil known from this lineage corresponds to a mid-Miocene †*C. hyomyzon* specimen. Thus, since several of the *Catostomus* fossils we review above (e.g. †*C. arenatus*, *C. discobolus*, and †*C. oromyzon*) are members of *Pantosteus* and dated to rocks younger than mid-Miocene in age, they were not used to calibrate *Pantosteus* nodes in BEAST.

**5. Additional Phylogenetic Relationships Within the Catostominae**

Within the Catostominae, ‘Clade 1’ (BPP = 1, most analyses) corresponded to the Thoburniini, including *Thoburnia* and *Hypentelium*. Relationships among *Moxostoma* lineages were generally unresolved; however, the concatenated mtDNA and mtDNA + morphology results congruently resolved *Moxostoma* sp. cf. *lachneri* from Surry County, in northwestern North Carolina (Great Pee Dee River), as sister to two clades with varying support that, with several exceptions, contained taxa associated with previously recognized subgenera *Scartomyzon* (‘Clade 2’; BPP = 0.74–1) and *Moxostoma* (‘Clade 3’; BPP = 0.55–0.96) (Figs. 1, S1 and S2). Alternatively, our four-locus and total-evidence topologies placed *M.* sp. cf. *lachneri* in Clade 3 with an unresolved position (Figs. 3 and S4). Subgenera *Moxostoma* and *Scartomyzon* were never resolved as monophyletic in Clade 2 or Clade 3. Within Clade 3, well-supported species groups included *M. anisurum* sister to *M. collapsum* (BPP = 1), *M. carinatum* 1 (Alabama) sister to *M.* sp. cf. *macrolepidotum* (BPP = 0.96–1), and *M. hubbsi* sister to the *M. breviceps* species group (BPP = 0.75–1) (Figs. 3, S1, S2 and S4). Alternative topologies placed *M. erythrurum* or *M. ariommum* sister to, or within, Clade 2, but with non-significant posterior support. With exceptions, relationships in Clade 2 generally collapse into a polytomy of four lineages: *M*. sp. cf. *poecilurum*; *M*. *duquesnei* sister to *M*. *poecilurum* + *M. congestum*; *M. carinatum* 2 (Minnesota) + *M. valenciennesi*; and the ‘*lachneri* group’, with *M. lachneri* sister to a clade of four Mexican suckers.

As an additional note on Clade 5, Unmack et al.’s (2014) analysis of *Pantosteus* also resolved *P. jordani*, *P. bondi*, and *P. lahontan* in a well supported clade based on mtDNA, and also showed that relationships in this clade appear not to have been influenced by introgressive hybridization events, based on comparing mtDNA and morphological characters. However, neither our study nor that of Doosey et al. (2010) sampled these lineages. Further sampling is thus required to see if these results would be supported by the nuclear loci we have examined, or by total-evidence dating analyses similar to those we employed herein.

Catostominae ‘Clade 6’ (BPP = 1; Fig. 3) contained species from basins on the eastern side of the Cascade and Sierra Mountain ranges in California, south-central Oregon, and northwestern Nevada, except for subspecies of *C. occidentalis* from the Central Valley and Pacific coast drainages of California. Subclade ‘6-a’ (BPP = 1) contained three species each restricted to a single drainage basin, including the Owens Valley sucker (*C. fumeiventris*) sister to a clade of Warner Valley (*C. warnerensis*) and Wall Canyon (*Catostomus* sp.) suckers (BPP = 1). In subclade ‘6-b’, four species from the Klamath Basin of south-central Oregon and northern California, including two species of *Catostomus*, *D. luxatus,* and *Chasmistes brevirostris*, formed a polytomy (BPP = 1). In subclade ‘6-c’, Modoc sucker (*C. microps*) were embedded within a clade containing four subspecies of *C. occidentalis* (BPP = 1).

Across analyses, we inferred a strongly supported sister relationship between a clade of *Catostomus catostomus* + *C. commersoni* (‘Clade 7’; BPP = 1) and a moderately well supported Catostominae ‘Clade 8’ + ‘Clade 9’ lineage composed of all remaining *Catostomus* (BPP = 0.71–91) (Figs. 3, S1, S2 and S4). Clade 8 contained species from the coastal rivers of Oregon, the Columbia River basin, the Great Basin, and Bonneville Basin resolved in a polytomy (BPP = 1). In the four-locus and IRBP analyses, which had the largest sample sizes we considered, *Chasmistes liorus mictus* and *C. ardens* from the Bonneville Basin were each inferred to be para-/polyphyletic in Clade 8 (Figs. S3 and S5). Also within Clade 8, *C. columbianus* from the Columbia River basin was sister to *C. tahoensis* from the Great Basin (BPP = 0.99–1). *Catostomus macrocheilus* from the Columbia River basin was resolved in a clade with *C. tsiltcoosensis* (Kettratad and Markle, 2010) and *Catostomus* sp. (Coquille River), both species from rivers of coastal Oregon; relationships among these taxa received definitive support (BPP = 1), except in the four-locus analysis where support varied (BPP = 0.79–0.99). ‘Clade 9’ contained species from the Colorado River basin, with *C. latipinnis* usually sister to a clade of *C. insignis* + *Xyrauchen texanus* in subclade ‘9-a’; a close affinity among members in this clade was strongly supported by analyses of the concatenated mtDNA, four-locus, and total-evidence datasets (BPP = 0.96–1) (Fig. 3). Four Mexican *Catostomus* species were placed in subclade ‘9-b’ with definitive support (BPP = 1); however, *C. bernardini* was polyphyletic in the concatenated mtDNA analysis, because specimens from the Río Batopilas formed a clade distinct from the Papagochic and Yecora rivers (Fig. S1).

**6. References**

Alvarado-Ortega J, Carranza-Castañeda O, Alvarez-Reyes G. 2006. A new fossil species of *Ictiobus* (Teleostei: Catostomidae) from Pliocene lacustrine sediments near Tula de Allende, Hidalgo, Mexico. *Journal of Paleontology* **80:**993-1008.

Armstrong RL, Leeman WP, Malde HE. 1975. K-Ar dating, Quaternary and Neogene volcanic rocks of the Snake River Plain, Idaho. *American Journal of Science* **275:**225-251.

Bell CJ, Lundelius Jr. E, Barnosky AD, Graham RW, Lindsay EH, Ruez Jr. DR, Semken Jr. HA, Webb SD, Zakrzewski RJ. 2004. The Blancan, Irvingtonian, and Rancholabrean Mammal Ages. In: Woodburne MO, ed. *Late Cretaceous and Cenozoic Mammals of North America: biostratigraphy and geochronology*. New York: Columbia University Press. 232-314

Betancur-RR, Broughton RE, Wiley EO, Carpenter K, López JA, Li C, Holcroft NI, Arcila D, Sanciangco M, Cureton JC, Zhang F, Buser T, Campbell MA, Ballesteros JA, Roa-Varon A, Willis S, Borden WC, Rowley T, Reneau PC, Lu G, Grande T, Arratia G, Ortí G. 2013. The tree of life and a new classification of bony fishes. *PLoS Currents Tree of Life*.

Broughton JM. 2000. Terminal Pleistocene fish remains from Homestead Cave, Utah, and implications for fish biogeography in the Bonneville Basin. *Copeia* **2000:**645-656.

Bruner JC. 1991. Comments on the genus *Amyzon* (family Catostomidae). *Journal of Paleontology* **65:**678-686.

Buth DG. 1980. Evolutionary genetics and systematic relationships in the catostomid genus *Hypentelium*. *Copeia* **1980:**280-290.

Cavender T. 1986. Review of the fossil history of North American freshwater fishes. In: Hocutt CH, Wiley EO, eds. *The zoogeography of North American freshwater fishes*. New York: John Wiley and Sons. 699-724

Cavender TM. 1998. Development of the North American Tertiary freshwater fish fauna with a look at parallel trends found in the European record. *Italian Journal of Zoology* **65(S1):**149-161.

Chang M-M, Chow CC. 1986. Stratigraphic and geographic distributions of the Late Mesozoic and Cenozoic fishes of China. In: Uyeno T, Arai R, Taniuchi T, Matsuura K, eds. *Indo-Pacific fish biology: proceedings of the second international conference on Indo-Pacific fishes*. Tokyo: The Ichthyological Society of Japan. 529-539

Chang M-M, Chen G. 2008. Fossil Cypriniformes from China and its adjacent areas and their palaeobiogeographical implications. In: Longbottom CL, Richter M, eds. *Fishes and the break-up of Pangaea*. London: Geological Society Special Publication 295. 337-350

Chang M, Miao D, Chen Y, Zhou J, Pingfu C. 2001. Suckers (Fish, Catostomidae) from the Eocene of China account for the family's current disjunct distributions. *Science China Series D-Earth Sciences* **44:**577-586.

Chen W-J, Mayden RL. 2012. Phylogeny of suckers (Teleostei: Cypriniformes: Catostomidae): further evidence of relationships provided by the single-copy nuclear gene IRBP2. *Zootaxa* **3586:**195-210.

Cheng CC. 1962. Fossil fishes from the early Teriary of Hsianghsiang, Hunan, with discussion of age of the Hsiawanpu formation (in Chinese with English Summary). *Vertebrata PalAsiatica* **6:**333-348.

Cope ED. 1872. On the Tertiary coal and fossils of Osino, Nevada. *Proceedings of the American Philosophical Society* **12:**478-481.

Cope ED. 1874. Report on the vertebrate paleontology of Colorado. *Annual report of U.S. geological and geographical survey of the terretories, embracing Colorado, 1873 (1874)* **2:**427-533.

Cope ED. 1875. On the fishes of the Tertiary shales of the South Park. *Bulletin of the U.S. Geological Survey, Survey of the Territories, 2^nd^ Series*, 3-5.

Cope ED. 1883. On the fishes of the recent and Pliocene lakes of the western part of the Great Basin, and of the Idaho Pliocene lake. *Proceedings of the Academy of Natural Sciences of Philadelphia* **35:**134-167.

Cope ED. 1893. Fossil fishes from British Columbia. *Proceedings of the Academy of Natural Sciences of Philadelphia* **45:**401-402.

Doosey M, Bart H, Saitoh K, Miya M. 2010. Phylogenetic relationships of catostomid fishes (Actinopterygii: Cypriniformes) based on mitochondrial ND4/ND5 gene sequences. *Molecular Phylogenetics and Evolution* **54:**1028-1034.

Eastman JT. 1977. The pharyngeal bones and teeth of catostomid fishes. *American Midland Naturalist* **97:**68-88.

Elder RL, Smith GR. 1988. Fish taphonomy and environmental inference in paleolimnology. Palaeogeogr. *Palaeoecology* **62:**577-592.

Ferris SD, Whitt GS. 1978. Phylogeny of tetraploid catostomid fishes based on the loss of duplicate gene expression. *Systematic Zoology* **27:**189-206.

Gavryushkina A, Welch D, Stadler T, Drummond AJ. 2014. Bayesian inference of sampled ancestor trees for epidemiology and fossil calibration. *PLoS Computational Biology* **10(12):**e1003919.

Gibbard PL, Head MJ, Walker MJC. The Subcommission on Quaternary Stratigraphy, 2009. Formal ratification of the Quaternary System/Period and the Pleistocene Series/Epoch with a base at 2.58 Ma. *Journal of Quaternary Science* **25(2):**96-102.

Grande L, Eastman JT, Cavender TM. 1982. *Amyzon gosiutensis*, a new catostomid fish from the Green River Formation. *Copeia* **1982:**523-532.

Greenwood DR, Archibald SB, Mathewes RW, Moss PT. 2005. Fossil biotas from the Okanagan Highlands, southern British Columbia and northeastern Washington state: climates and ecosystems across an Eocene landscape. *Canadian Journal of Earth Science* **42:**167-185.

Harris PM, Hubbard G, Sandel M. 2014. Catostomidae: Suckers. In: Warren ML, Burr BM, eds. *Freshwater fishes of North America: Volume 1: Petromyzontidae to Catostomidae.* Baltimore, MD: Johns Hopkins University Press. 451-502

Harris PM, Mayden RL. 2001. Phylogenetic relationships of major clades of Catostomidae (Teleostei: Cypriniformes) as inferred from mitochondrial SSU and LSU rDNA sequences. *Molecular Phylogenetics and Evolution* **20:**225-237.

Harris PM, Mayden RL, Perez HE, Garcia de Leon F. 2002. Phylogenetic relationships of Redhorse (*Moxostoma*) and Jumprock (*Scartomyzon*) suckers (Cypriniformes: Catostomidae) based on mitochondrial cytochrome *b* sequence data. *Journal of Fish Biology* **61:**1433-1452.

Heath TA, Huelsenbeck JP, Stadler T. 2014. The fossilized birth–death process for coherent calibration of divergence-time estimates. *Proceedings of the National Academy of Sciences of the United States of America* **111:**E2957-E2966.

Hibbard CW. 1963. A Late Illinoian fauna from Kansas and its climatic significance. *Papers of the Michigan Academy of Science, Arts & Letters* **VLVIII 1963 (1962):**187-221.

Hoetker GM, Gobalet KW. 1999. Fossil razorback sucker (Pisces: Catostomidae, *Xyrauchen texanus*) from southeastern California. *Copeia* **1999:**755-759.

Ho SYW, Phillips MJ. 2009. Accounting for calibration uncertainty in phylogenetic estimation of evolutionary divergence times. *Systematic Biology* **58:**367-380.

Hussakof L. 1932. The fossil collected by the Central Asiatic expedition. *American Museum Novitates* **553:**1-19.

Israde-Alcántara I, Miller WE, Garduño-Monroy VH, Barron J, Rodriguez-Pascua MA. 2010. Palaeoenvironmental significance of diatom and vertebrate fossils from Late Cenozoic tectonic basins in west-central México: a review. *Quaternary International* **219:**79-94.

Liu J, Chang M. 2009. A new Eocene catostomid (Teleostei: Cypriniformes) from northeastern China and early divergence of Catostomidae. *Science China Series D: Earth Sciences* **52:**189-202.

Markewich HW, Wysocki DA, Pavich MJ, Rutledge EM. 2011. Age, genesis, and paleoclimatic interpretation of the Sangamon/Loveland complex in the Lower Mississippi Valley, U.S.A. *Geological Society of America Bulletin* **123:**21-39.

Marshall CR. 1990. Confidence intervals on stratigraphic ranges. *Paleobiology* **16:**1-10.

Mauger RL. 1977. K-Ar ages of biotites from the tuffs in Eocene rocks of the Green River, Washakie, and Uinta Basins, Wyoming, and Colorado. *University of Wyoming Contributions to Geology* **15:**17-41.

Miller RR, Smith GR. 1967. New fossil fishes from Plio–Pleistocene Lake Idaho. *Occasional Papers of the Museum of Zoology, University of Michigan* **654:**1-24.

Minckley WL. 1973. *Fishes of Arizona.* Phoenix, AZ: Arizona Game and Fish Department.

Near TJ, Bolnick DI, Wainwright PC. 2005. Fossil calibrations and molecular divergence time estimates in centrarchid fishes (Teleostei: Centrarchidae). *Evolution* **59:**1768-1782.

Near TJ, Bossu CM, Bradburd GS, Carlson RL, Harrington RC, Hollingsworth Jr. PR, Keck BP, Etnier DA. 2011. Phylogeny and temporal diversification of darters (Percidae: Etheostomatinae). *Systematic Biology* **60:**565-595.

Near TJ, Eytan RI, Dornburg A, Kuhn KL, Moore JA, Davis MP, Wainwright PC, Friedman M, Smith, WL. 2012. Resolution of ray-finned fish phylogeny and timing of diversification. *Proceedings of the National Academy of Sciences of the United States of America U.S.A.* **109:**13698-13703.

Neff NA. 1975. Fishes of the Kanopolis local fauna (Pleistocene) of Ellsworth County, Kansas. In: Friedland NE, Smith GR, eds. *Studies on Cenozoic paleontology and stratigraphy: symposium held at the University of Michigan, May 6 and 7, 1974; in Honor of Claude W. Hibbard*. *Papers on Paleontology the Museum of Paleontology, University of Michigan* **12:** 39-48.

Nelson EM. 1949. The opercular series of the Catostomidae. *Journal of Morphology* **85:**559-567.

Parham JF, Donoghue PCJ, Bell CJ, Calway TD, Head JJ, Holroyd PA, Inoue JG, Irmis RB, Joyce WG, Ksepka DT, Patané JSL, Smith ND, Tarver JE, van Tuinen M, Yang Z, Angielczyk KD, Greenwood JM, Hipsley CA, Jacobs L, Makovicky PJ, Müller J, Smith KT, Theodor JM, Warnock RCM, Benton MJ. 2012. Best practices for justifying fossil calibrations. *Systematic Biology* **61:**346-359.

Prothero DR, Sanchez F. 2004. Magnetic stratigraphy of the Upper Eocene Florissant Formation, Teller County, Colorado. In: Lucas SG, Zeigler KE, Kondrashov PE, eds. *Paleogene mammals*. *New Mexico Museum of Natural History and Science Bulletin* **26:**145-149.

Prothero DR, Swisher CCI. 1992. Magnetostratigraphy and geochronology of the terrestrial Eocene–Oligocene transition in North America. In: Prothero DR, Berggren WA, eds. *Eocene–Oligocene climatic and biotic evolution*. Princeton, NJ: Princeton University Press. 74-87

Pyron RA. 2011. Divergence time estimation using fossils as terminal taxa and the origins of Lissamphibia. *Systematic Biology* **60:**466-481.

Repenning CA. 1987. Biochronology of the microtine rodents of the United States. In: Woodburne MO, ed. *Cenozoic mammals of North America*. Berkeley, CA: University of California Press. 236-268

Reynolds RE, Lindsay EH. 1999. Late Tertiary basins and vertebrate faunas along the Nevada-Utah border. In: Gillette DD, ed. *Vertebrate pale­ontology in Utah*. *Utah Geological Survey Miscellaneous Publication* **99-1:**469-478.

Ruez Jr. DR, Ginsler PA. 2008. An unexpected early record of *Mictomys vetus* (Arvicolinae, Rodentia) from the Blancan (Pliocene) Glenns Ferry Formation, Hagerman Fossil Beds National Monument, Idaho. *Journal of Paleontology* **82:**650-654.

Schmidt DL, Page WR, Workman JB. 1996. Preliminary geological map of the Moapa West Quadrangle, Clark County, Nevada. *U.S.G.S. Open-File Report 96-521*.

Smith CL. 1954. Pleistocene fishes of the Berends fauna of Beaver County, Oklahoma. *Copeia* **1954:**282-289.

Smith CL. 1962. Some Pliocene fishes from Kansas, Oklahoma, and Nebraska. *Copeia* **1962:**505-520.

Smith GR. 1963. A Late Illinoian fish fauna from southwestern Kansas and its climatic significance. *Copeia* **1963:**278-285.

Smith GR. 1966. Distribution and evolution of the North American catostomid fishes of the subgenus *Pantosteus*, genus *Catostomus*. *Miscellaneous Publications of the Museum of Zoology, University of Michigan* **129:**1-132.

Smith GR. 1975. Fishes of the Pliocene Glenns Ferry formation, southwest Idaho. *University of Michigan Museum of Paleontology, Papers on Paleontology* **5:**1-68.

Smith GR. 1981. Late Cenozoic freshwater fishes of North America. *Annual Review of Ecology and Systematics* **12:**163-193.

Smith GR. 1992. Phylogeny and biogeography of the Catostomidae, freshwater fishes of North America and Asia. In: Mayden RL, ed. *Systematics, historical ecology, and North American freshwater fishes.* Stanford, CA: Stanford University Press. 778-813

Smith GR, Badgley CE, Eiting TP, Larson PS. 2010. Species diversity gradi­ents in relation to geological history in North American freshwater fishes. *Evolutionary Ecology Research* **12:**693-726.

Smith GR, Dowling TE, Gobalet KW, Lugaski T, Shiozawa DK, Evans RP. 2002. Biogeography and timing of evolutionary events among Great Basin fishes. In: Hershler R et al., eds. *Great Basin aquatic systems history*. Washington, D.C.: Smithsonian Institution Press. 175-234

Smith GR, Koehn RK. 1971. Phenetic and cladistic studies of biochemical and morphological characteristics of *Catostomus*. *Systematic Zoology* **20:**282-297.

Smith GR, Lundberg JG. 1972. The Sand Draw fish fauna. In: Skinner MF, Hibbard CW, eds. *Pleistocene preglacial and glacial rocks and faunas of north central Nebraska.* New York: American Museum of Natural History. 40-54

Smith GR, Stewart JD, Carpenter NE. 2013. Fossil and recent mountain suckers *Pantosteus*, and significance of introgression in catostomin fishes of Western United States. *Occasional Papers of the Museum of Zoology, University of Michigan* **724:**1-59.

Smith GR, Swirydczuk K, Kimmel PG, Wilkinson BH. 1982. Fish biostratig­raphy of late Miocene to Pleistocene sediments of the western Snake River Plain, Idaho. In: Bonnichsen B, Breckenridge RM, eds. *Cenozoic geol­ogy of Idaho*. Idaho Bureau of Mines and Geology Bulletin **26:**519-542.

Smith KS, Cifelli RL. 2000. A synopsis of the Pleistocene vertebrates of Oklahoma. *Oklahoma Geological Survey Bulletin* **147:**1-36.

Smith ML, Cavender TM, Miller RR. 1975. Climatic and biogeographic significance of a fish fauna from the Late Pliocene-Early Pleistocene of the Lake Chapala Basin (Jalisco, Mexico). In: Friedland NE, Smith GR, eds. *Studies on Cenozoic paleontology and stratigraphy: symposium held at the University of Michigan, May 6 and 7, 1974; in Honor of Claude W. Hibbard*. *Papers on Paleontology the Museum of Paleontology University of Michigan* **12:**29-38.

Steward JD, Roeder MA. 1993. Razorback sucker (*Xyrauchen texanus*) fossils from the Anza-Borrego Desert and the ancestral Colorado River. Redlands, CA: San Bernardino County Museum Association Special Publication 93.

Sun YH, Xie CX, Wang WM, Liu SY, Treer T, Chang MM. 2007. The genetic variation and biogeography of catostomid fishes based on mitochondrial and nucleic DNA sequences. *Journal of Fish Biology* **70:**291-309.

Swisher III CC, Prothero DR. 1990. Single-crystal ^40^Ar/^39^Ar dating of the Eocene–Oligocene transition in North America. *Science* **249:**760-762.

Sytchevskaya EK. 1986. Paleogene freshwater fish fauna of the USSR and Mongolia (in Russian). *Joint Soviet-Mongolian Palaeontological Expedition Transactions* **39:**77-82.

Taylor DW, Smith GR. 1981. Pliocene molluscs and fishes from northeastern California and northwestern Nevada. *Contributions from the Museum of Paleontology, the University of Michigan* **25:**339-413.

Tedford RH, Albright III LB, Barnosky AD, Ferrusquía-Villafranca I, Hunt Jr. RM, Storer JE, Swisher III CC, Voorhies MR, Webb SD, Whistler DP. 2004. Mammalian biochronology of the Arikareean through Hemphillian interval (Late Oligocene through Early Pliocene epochs). In: Woodburne MO, ed. *Late Cretaceous and Cenozoic mammals of North America: biostratigraphy and geochronology.* New York: Columbia University Press. 232-314

Tedford RH, Skinner MF, Fields RW, Rensberger JM, Whistler DP, Galusha T, Taylor BE, Macdonald JR, Webb SD. 1987. Faunal succession and the biochronology of the Arikareean through Hemphillian interval (Late Oligocene through earliest Pliocene epochs) in North America. In: Woodburne MO, ed. *Cenozoic mammals of North America.* Berkeley, CA: University of California Press. 153-210

Unmack PJ, Dowling TE, Laitinen NJ, Secor CL, Mayden RL, Shiozawa DK, Smith GR. 2014. Influence of introgression and geological processes on phylogenetic relationships of western North American mountain suckers (*Pantosteus*, Catostomidae). *PLoS One* **9:**e90061.

Uyeno T, Miller RR. 1962. Late Pleistocene fishes from a Trinity River Terrace, Texas. *Copeia* **1962:**338-345.

Wang BY, Li CT. 1990. First Paleogene mammalian fauna from northeastern China (in Chinese with English summary). *Vertebrata PalAsiatica* **28(3):**165-205.

Walker JD, Geissman JW, Bowring SA, Babcock LE. (compilers) 2012. Geologic time scale v. 4.0. Boulder, CO: Geological Society of America.

Wilson MVH. 1977. Middle Eocene freshwater fishes from British Columbia. *Life Sciences Contributions 113, Royal Ontario Museum, Toronto, Ontario.* 1-61

Wilson RL. 1968. Systematics and faunal analysis of a Lower Pliocene vertebrate assemblage from Trego County, Kansas. *Contributions from the Museum of Plaeontology, the University of Michigan* **22:**75-126.

Wolfe JA, Gregory-Wodzicki KM, Molnar P, Mustoe G. 2003. Rapid uplift and then collapse in the Eocene of the Okanagan? Evidence from paleobotany. *Geological Association of Canada – Mineralogical Association of Canada – Society of Economic Geologists, Joint Annual Meeting, (Vancouver, Canada) Abstracts 28*, abstract 533.

Wood JR, Forman SL, Pierson J, Gomez J. 2010. New insights on Illinoian deglaciation from deposits of Glacial Lake Quincy, central Indiana. *Quaternary Research* **73:**374-384.

Woodburne MO. 2007. Phyletic diversification of the *Cormohipparion occidentale* complex (Mammalia: Perissodactyla, Equidae), Late Miocene, North America, and the origin of the Old World *Hippotherium* datum. *Bulletin of the American Museum of Natural History* **306:**1-138.
