# Supplementary material for: Phylogeny and divergence times of suckers (Cypriniformes: Catostomidae) inferred from Bayesian total-evidence analyses of molecules, morphology, and fossils
Source: PeerJ. 2018 Jul 4;6:e5168. doi: 10.7717/peerj.5168 (PMC6035723; doi:10.7717/peerj.5168)
Supplement: Supplemental Information 2 — Model selection analyses using PartitionFinder v1.1.1 supported different best-fit models of DNA evolution for different data subsets (i.e. groups or ‘blocks’ of data, such as sites filtered by codon positions). When it was not possible to specify a given model in phylogenetic software, we used the next most closely related model in the GTR family of models. Symbols and abbreviations: Γ, gamma-distributed rate variation; bp, number of nucleotide base pairs; I, parameter representing proportion of invariable sites; n, sample size (numbers correspond to sequence alignment sizes, except for multilocus datasets the numbers in parentheses are sample sizes for each locus); no., subset number. [file peerj-06-5168-s002.docx]

| **No.** | **Subset** | ***n*** | **bp** | **Best model** |
| --- | --- | --- | --- | --- |
| 1 | mtDNA (cyt*b*, ND2, and *cox1*) 1^st^ codon position | 126(102) | 946 | TVM+*Γ+I* |
| 2 | mtDNA (cyt*b*, ND2, and *cox1*) 2^nd^ codon position | 126(102) | 945 | GTR+*Γ+I* |
| 3 | mtDNA (cyt*b*, ND2, and *cox1*) 3^rd^ codon position | 126(102) | 945 | GTR+*Γ+I* |
| 4 | nuclear IRBP 1^st^ codon position | 113(102) | 279 | TIM+*Γ+I* |
| 5 | nuclear IRBP 2^nd^ codon position | 113(102) | 280 | K80+*Γ* |
| 6 | nuclear IRBP 3^rd^ codon position | 113(102) | 280 | TVMef+*Γ* |
| 7 | nuclear GHI + *RPS7* sites, combined | 113+52(102) | 2250 | K81uf+*Γ* |
