# Supplementary figures and images for: Phylogeny and divergence times of suckers (Cypriniformes: Catostomidae) inferred from Bayesian total-evidence analyses of molecules, morphology, and fossils
Source: PeerJ. 2018 Jul 4;6:e5168. doi: 10.7717/peerj.5168 (PMC6035723; doi:10.7717/peerj.5168)

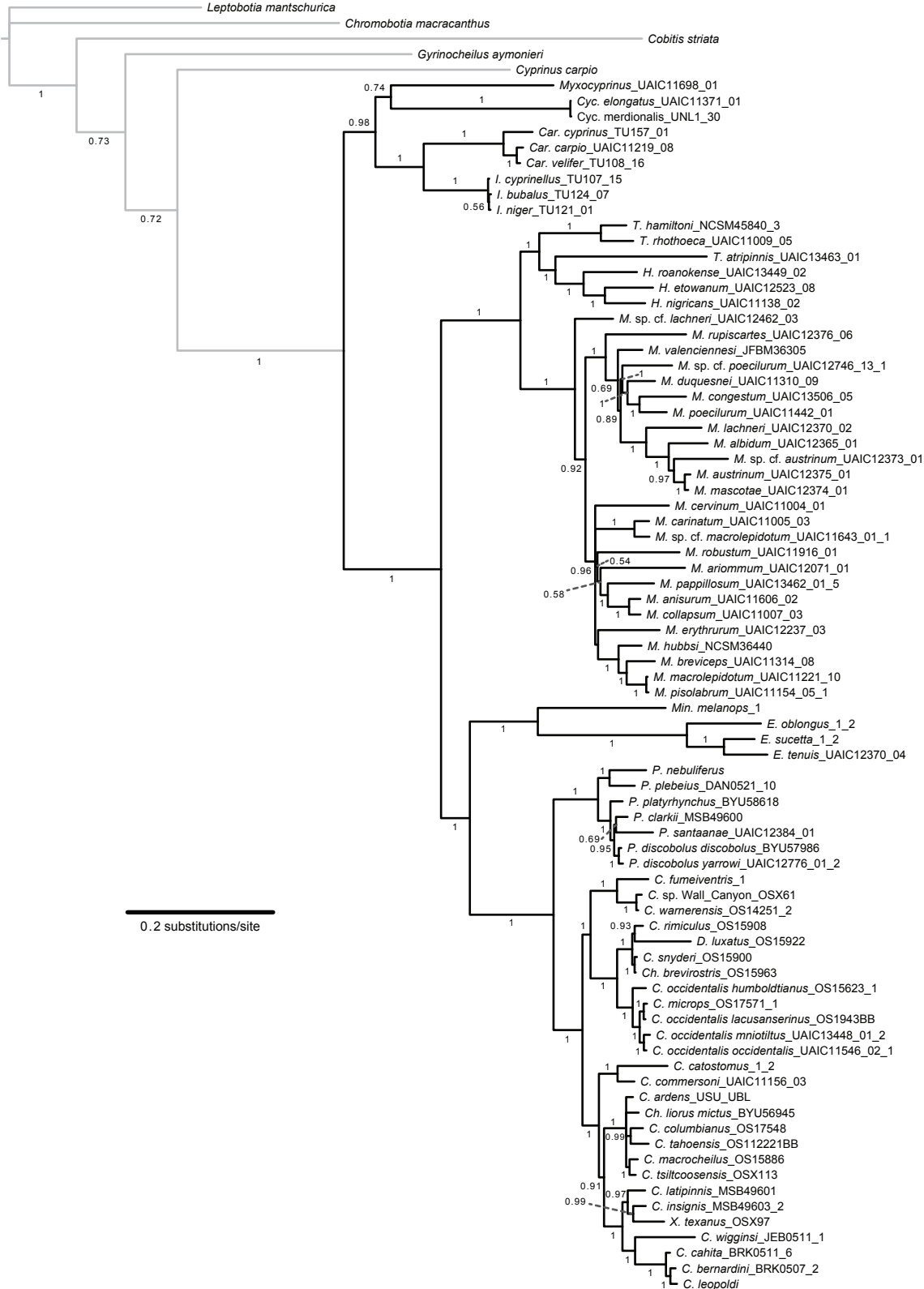

Supplement: Supplemental Information 3 [file peerj-06-5168-s003.pdf]

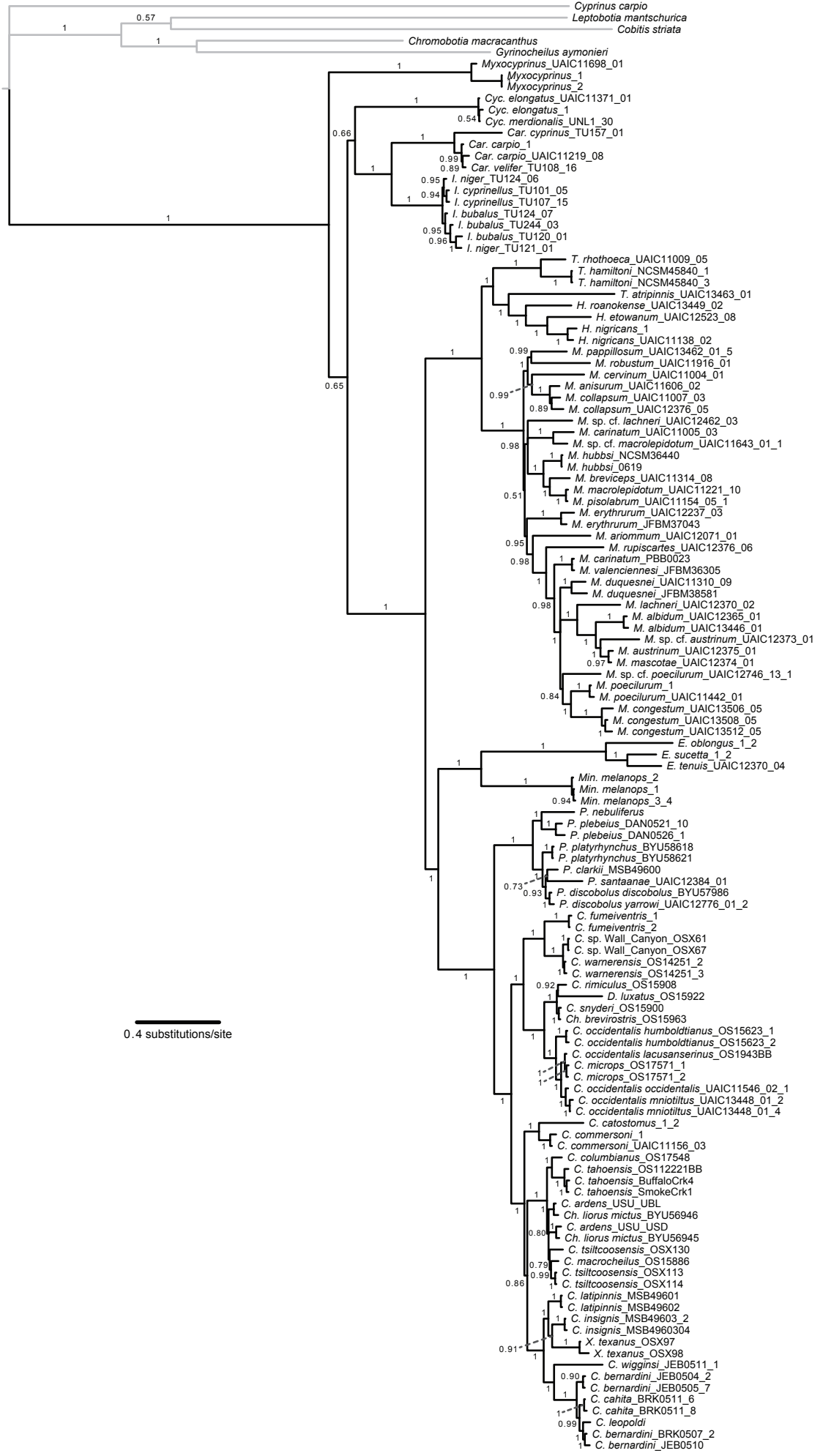

Supplement: Supplemental Information 4 [file peerj-06-5168-s004.pdf]

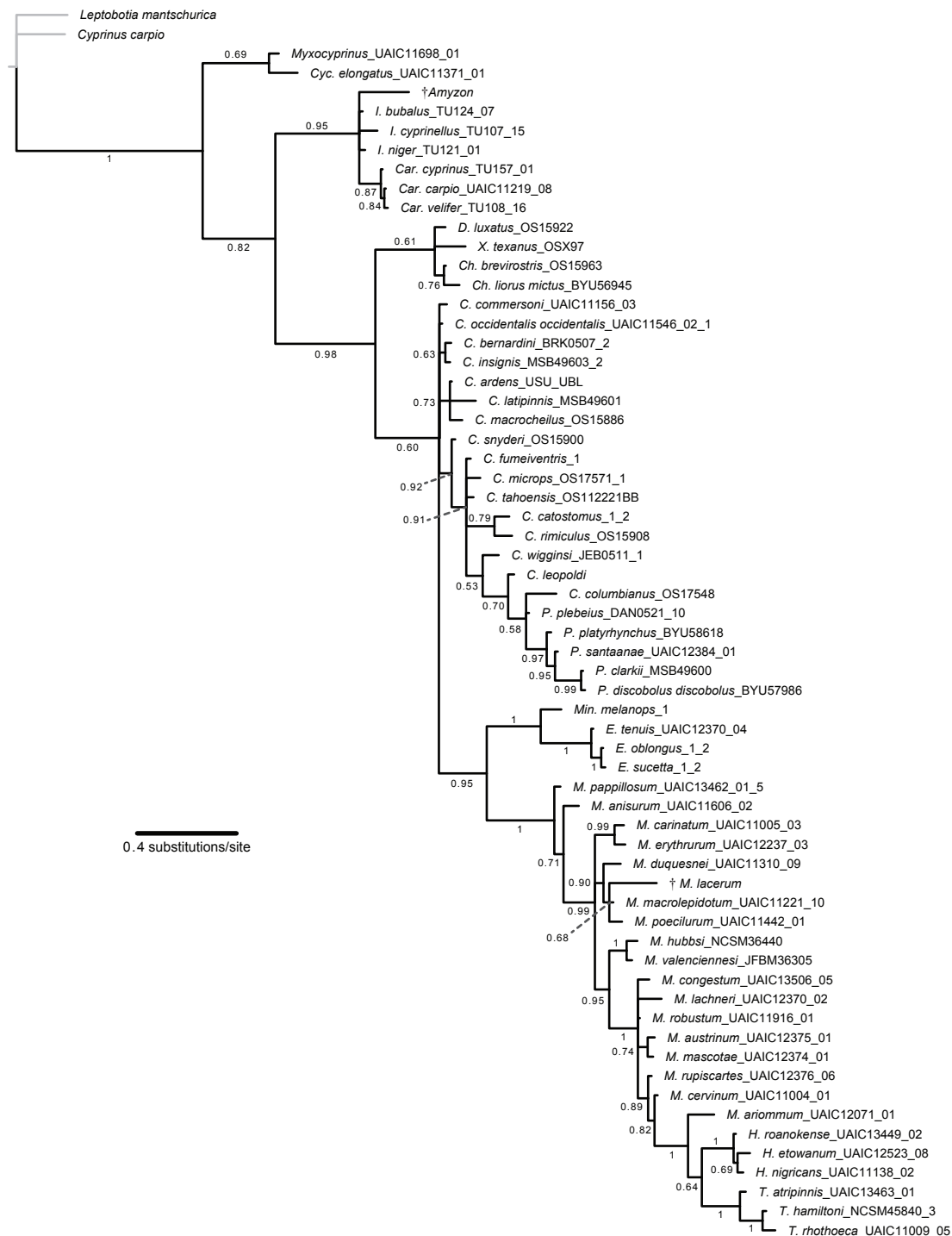

Supplement: Supplemental Information 5 [file peerj-06-5168-s005.pdf]

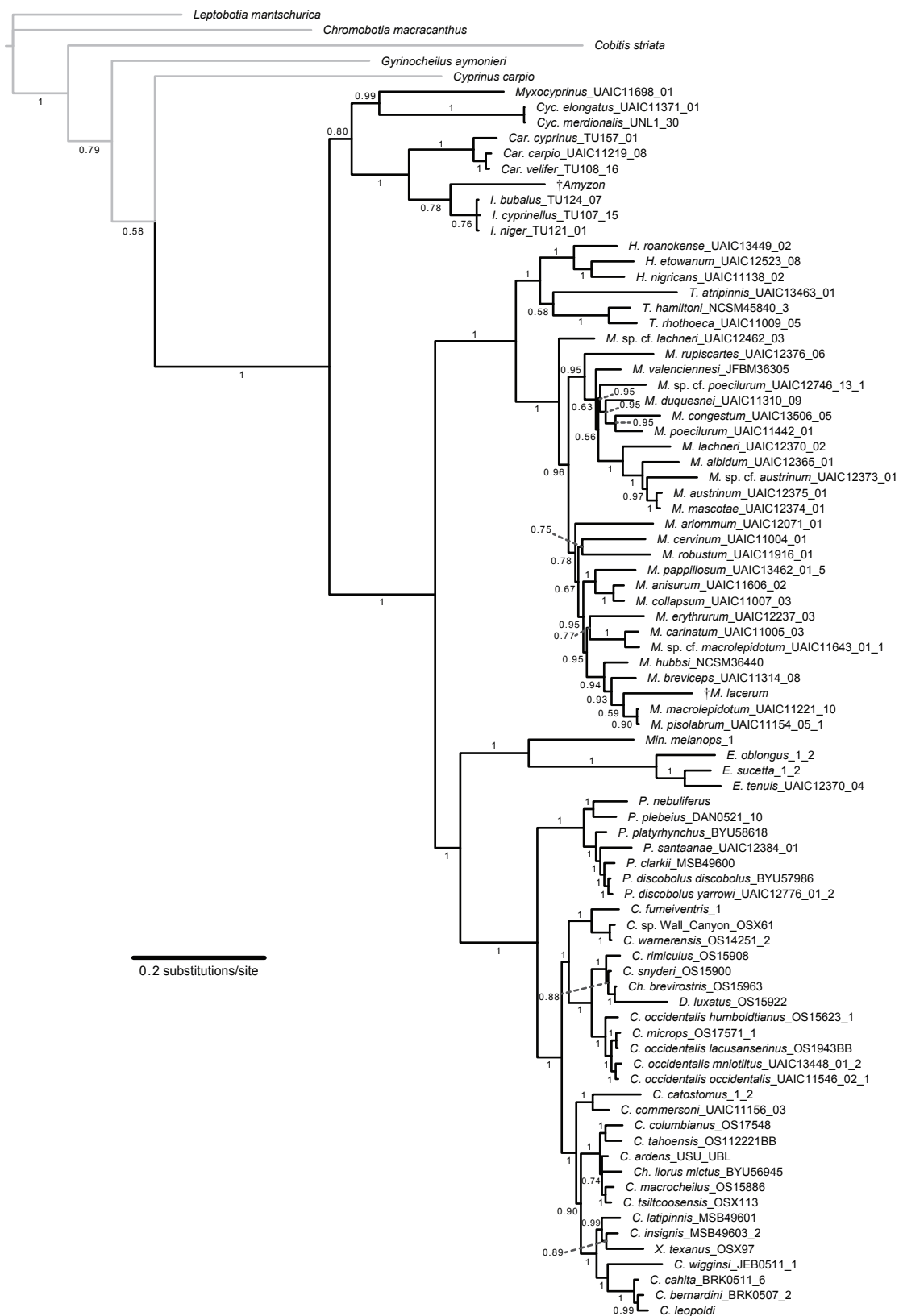

Supplement: Supplemental Information 6 [file peerj-06-5168-s006.pdf]

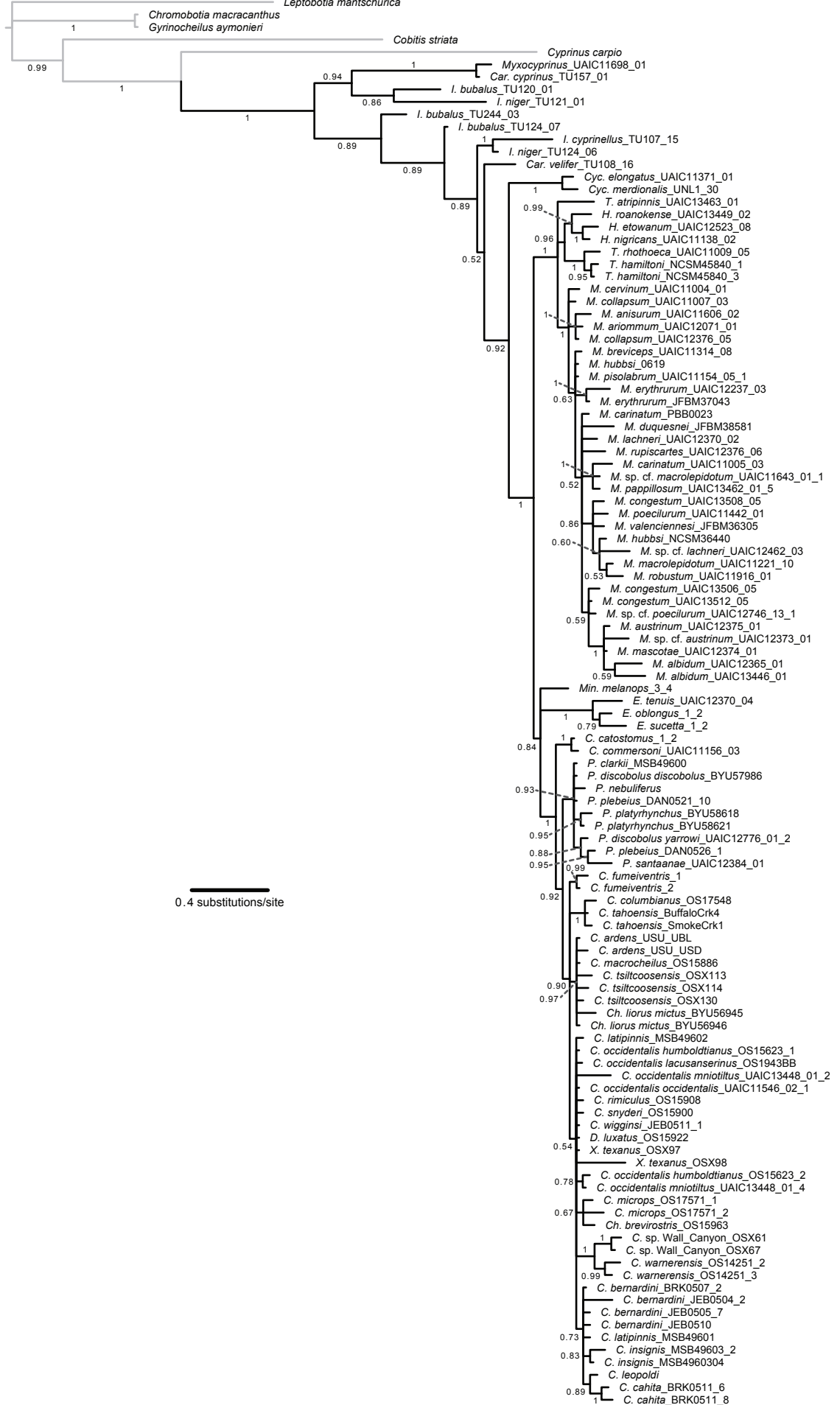

Supplement: Supplemental Information 7 [file peerj-06-5168-s007.pdf]

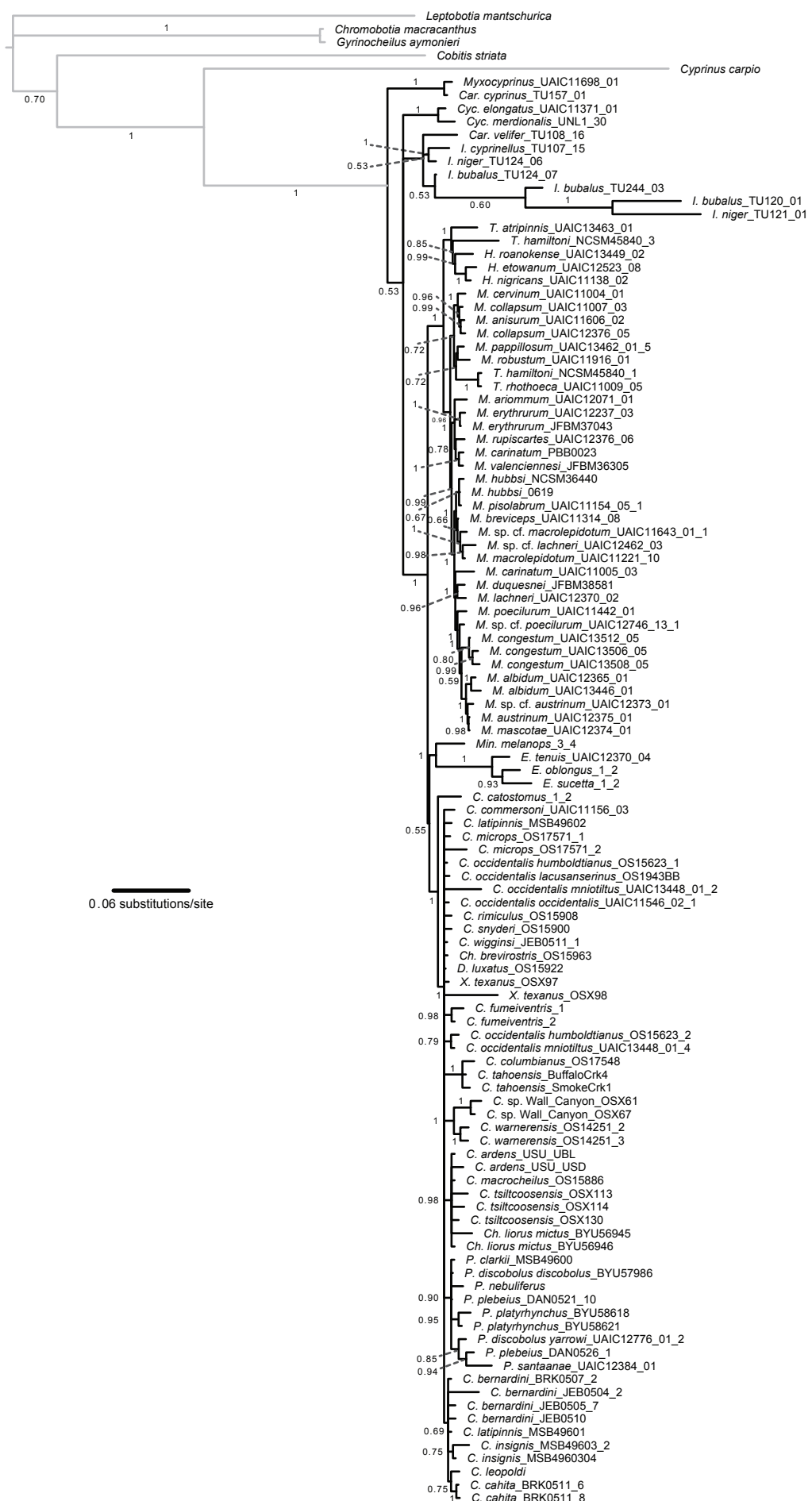

Supplement: Supplemental Information 8 [file peerj-06-5168-s008.pdf]

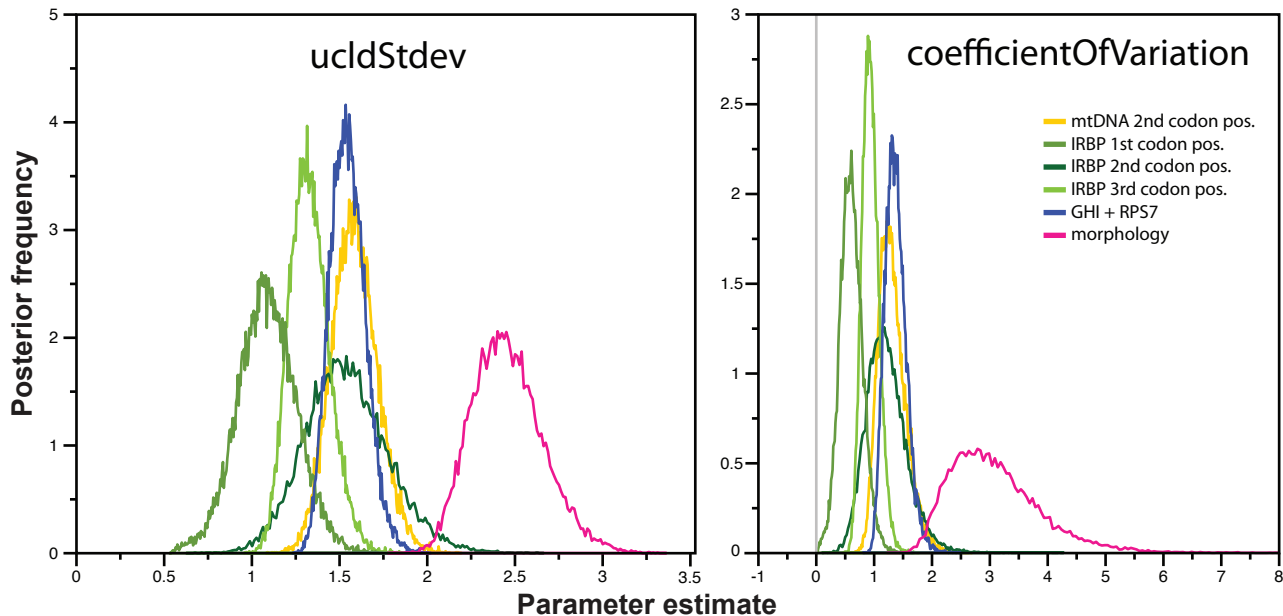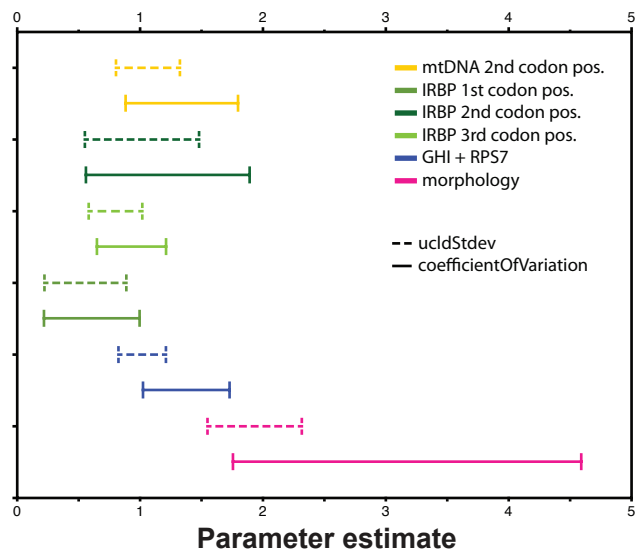

Supplement: Supplemental Information 9 — Problematic mtDNA 1st and 3rd codon sites were excluded from the analysis. [file peerj-06-5168-s009.pdf]

DNA partition  $\Gamma$  shape parameter

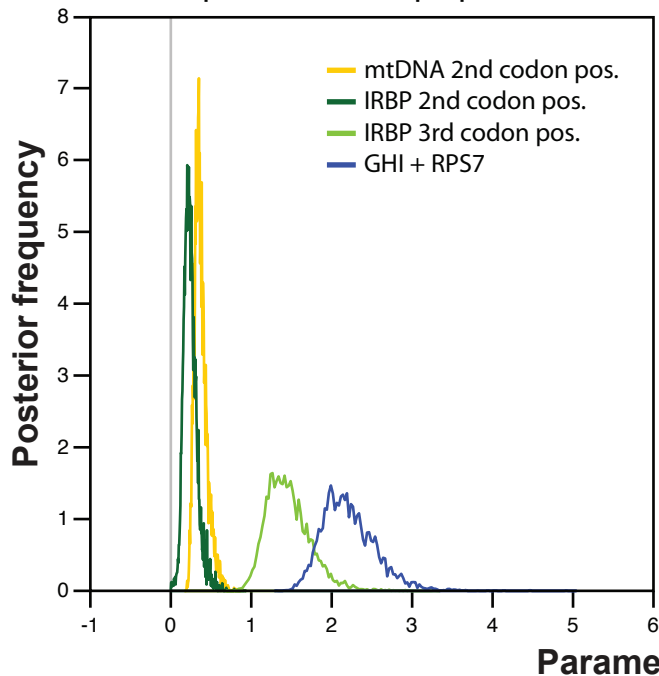

morphology partition  $\Gamma$  shape parameter

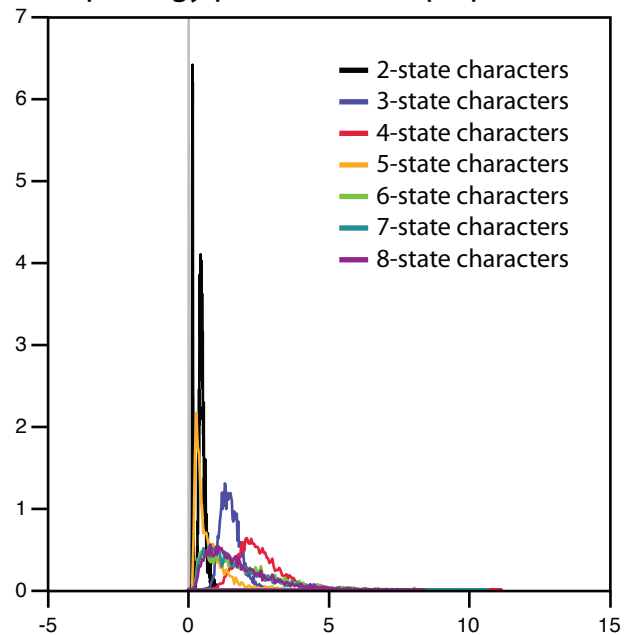

Supplement: Supplemental Information 10 — This figure plots variation in substitution rates among sites, based on posterior distributions of the gamma shape parameters (alpha estimates) assigned to different data subsets in BEAST (excluding problematic mtDNA 1stand 3rd codon position sites). [file peerj-06-5168-s010.pdf]

Probability correct

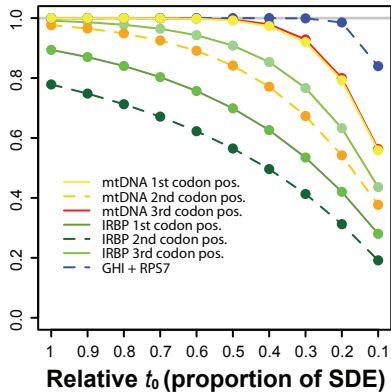

Probability polytomy

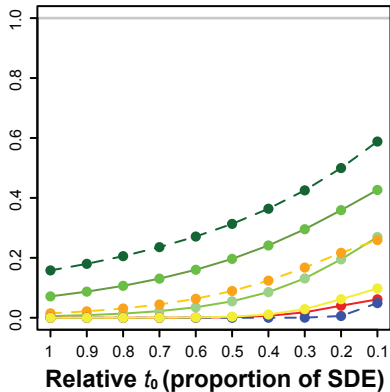

Probability Incorrect

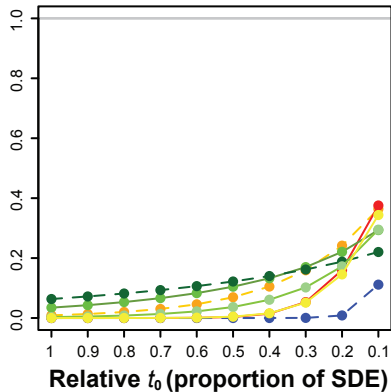

Supplement: Supplemental Information 11 — The period investigated is the ‘subfamily divergence epoch’ (SDE; ∼63.16–34.37 Ma) in our time tree. The SDE defines an internode distance, t0, used in the probability approximations. Results are shown across relative t0 values reflecting t0 proportions declining from 100% (1) to 10% (0.1) along the x-axis. [file peerj-06-5168-s011.pdf]
